# Loss of *msnA*, a Putative Stress Regulatory Gene, in *Aspergillus parasiticus *and *Aspergillus flavus* Increased Production of Conidia, Aflatoxins and Kojic Acid

**DOI:** 10.3390/toxins3010082

**Published:** 2011-01-12

**Authors:** Perng-Kuang Chang, Leslie L. Scharfenstein, Meng Luo, Noreen Mahoney, Russell J. Molyneux, Jiujiang Yu, Robert L. Brown, Bruce C. Campbell

**Affiliations:** 1 Southern Regional Research Center, Agricultural Research Service, U. S. Department of Agriculture, 1100 Robert E. Lee Boulevard, New Orleans, LA 70124, USA; Email: les.scharfenstein@ars.usda.gov (L.L.S.); meng.luo@ars.usda.gov (M.L.); jiujiang.yu@ars.usda.gov (J.Y.); robert.brown@ars.usda.gov (R.L.B.); 2 Western Regional Research Center, Agricultural Research Service, U. S. Department of Agriculture, 800 Buchanan Street, Albany, CA 94710, USA; Email: noreen.mahoney@ars.usda.gov (N.M.); molyneux@hawaii.edu (R.J.M.); molyneux@hawaii.edu (B.C.C.); 3 Current adddress: College of Pharmacy, University of Hawaii at Hilo, 200 W. Kawili Street, Hilo, HI 96720, USA

**Keywords:** *Aspergillus*, aflatoxin, kojic acid, oxidative stress, development

## Abstract

Production of the harmful carcinogenic aflatoxins by *Aspergillus parasiticus* and *Aspergillus flavus* has been postulated to be a mechanism to relieve oxidative stress. The *msnA* gene of *A. parasiticus* and *A. flavus* is the ortholog of *Saccharomyces cerevisiae MSN2* that is associated with multi-stress response. Compared to wild type strains, the *msnA *deletion (∆msnA) strains of *A. parasiticus* and *A. flavus* exhibited retarded colony growth with increased conidiation. The ∆msnA strains also produced slightly higher amounts of aflatoxins and elevated amounts of kojic acid on mixed cereal medium. Microarray assays showed that expression of genes encoding oxidative stress defense enzymes, *i.e.*, superoxide dismutase, catalase, and cytochrome c peroxidase in *A. parasiticus* ∆msnA, and the catalase A gene in *A. flavus* ∆msnA, was up-regulated. Both *A. parasiticus* and *A. flavus* ∆msnA strains produced higher levels of reactive oxygen species (ROS), and ROS production of *A. flavus* msnA addback strains was decreased to levels comparable to that of the wild type *A. flavus*. The *msnA* gene appears to be required for the maintenance of the normal oxidative state. The impairment of *msnA* resulted in the aforementioned changes, which might be used to combat the increased oxidative stress in the cells.

## 1. Introduction

In nature all living organisms react to unfavorable environmental conditions, such as high temperature, osmotic shock, oxidative damage and nutrient depletion via complex regulatory networks. These specific responses usually result from induction of a set of stress-associated genes whose expression is controlled by a common transcription factor. For example, in *Saccharomyces cerevisiae* a gene named *MSN2* that encodes a C_2_H_2_-type zinc-finger regulator, Msn2p, is required for yeast cells to cope with a broad range of environmental and physiological stresses [1]. Msn2p mediates expression of a number of genes that are induced by stress conditions by binding to STRE (stress response element) motifs, CCCCT, which are located in the promoters of the regulated genes [2,3]. In *Trichoderma atrovirde* the expression of the *MSN2* ortholog *seb1 *(stress response element binding) was increased under osmotic stress conditions [4]. Seb1 functions to up-regulate the glycerol dehydrogenase gene (*gld1*) whose product, Gld1, is required for glycerol biosynthesis to alleviate osmotic stress [5].

Conidiation and formation of sclerotia, hyphal aggregates that serve as the over-winter structure, are believed to be triggered by a hyperoxidant state (oxidative stress) in cells at late stages of fungal development [6,7]. These processes are often interrelated with production of secondary metabolites, such as aflatoxins [8,9,10] which may allow fungi to adapt to unique niches and life cycles. Jeon *et al.* [8] showed that deletion of the *MSN2* ortholog (*msnA*) in *Aspergillus nidulans *resulted in enhanced asexual conidiation and production of sexual cleistothecia. Hence, they renamed the *A. nidulansmsnA* as *nrdA* (negative regulator of differentiation). Not all *Aspergillus* species have a natural sexual reproductive stage. *Aspergillus parasiticus* and *Aspergillus flavus*, the predominant producers of the aflatoxins, were thought, until recently, to possess only the asexual state, but under forced mating conditions in the laboratories strains of opposite mating types were able to undergo sexual reproduction [9,10]. The majority of *A. parasiticus* strains produce abundant conidia, but some strains, in addition to conidia, also produce large numbers of sclerotia on the same media [11]. *A. flavus* strains are morphologically diverse. Some strains produce predominantly conidia with a few large-sized sclerotia, and others produce copious tiny sclerotia along with a low number of conidia; the former is called L-strain and the latter S-strain [12]. 

In this study, we investigated the role of *msnA* in two morphologically distinct *A. parasiticus *strains and an L-strain *A. flavus* isolate. Deletion of *msnA* adversely affected vegetative growth and altered development as manifested by dense conidiation and the lack of sclerotial formation. Expression of oxidative stress defense genes and the production of kojic acid (5-hydroxy-2-(hydroxymethyl)-4-pyrone), a free radical scavenger, increased significantly in the *A. parasiticus* ∆msnA strains. Compared to respective wild-type strains, the ∆msnA strains of *A. parasiticus* and *A. flavus* produced increased levels of reactive oxygen species. 

## 2. Materials and Methods

### 2.1. Fungal Strains and Media

*Aspergillus parasiticus *BN9∆ku70 [13] and RH∆ku70 [14] and *A. flavus* CA14PTs∆pyrG [15], a ∆ku70 strain sensitive to pyrithiamine, were the recipient strains used in the *msnA* gene knockout experiments. *A. parasiticus* BN9∆ku70 and *A. flavus* CA14PTs∆pyrG are aflatoxigenic and produce abundant conidia and a few sclerotia. RH∆ku70 accumulates *O*-methylsterigmatocystin (OMST) as the end product; it produces abundant sclerotia and conidia when grown in the dark. OMST-accumulating isolates have been found to account for about 2.6% of an *A. parasiticus* population in a southwestern Georgia peanut field [11]. Potato Dextrose Agar (PDA; EMD Chemicals Inc., Darmstadt, Germany) was used for fungal growth and production of conidia and sclerotia for enumeration. The mixed cereal agar (MCA, 5% Gerber^®^ Mixed Grain Cereal, 1.5% agar) [16] was also used to promote sclerotial production. 

### 2.2. Construction of the *msnA* Disruption Vector

TheEST of the *A. flavus msnA* gene (NAFAE55TH) was identified initially from the *Aspergillus flavus* Gene Index database at The Institute for Genomic Research (TIGR) based on *S. cerevisiaeMSN2* and its homologue in *A. nidulans* (AN1652.2). The complete *A. flavus msnA* gene was subsequently obtained from the *Aspergillus* Comparative Database at Broad Institute (http://www.broadinstitute.org/annotation/genome/aspergillus_group/MultiHome.html). Restriction analysis of *A. flavus msnA *and flanking regions was carried out using the DNAMAN software (Lynnon Soft, Vandreuil, Quebec, Canada). The *msnA* disruption vector was constructed as follows: a 1.4-kb *msnA* 5’ and coding region and a 0.7-kb coding region near the 3’ end were amplified by PCR using primers msn5K: CTGTCTCCCGGTACCTTTGATCG and msn5P: GAGTATGCGCTGCAGCGCTGTCTC, and msn3P: GAGACAGCGCTGCAGCGCATACTC and msn3H: CGTGGGAAGCTTCATAGAGCAC, respectively. The PCR fragments after digestion were cloned sequentially into corresponding sites in pUC19. The 2.0-kb *A. oryzaeptr* selectable marker amplified from pPTR1 [17] was cloned into the PstI site of the above construct. This disruption vector construct, msnDV, was linearized by HindIII and KpnI to release the portion of pUC19 prior to fungal transformation.

### 2.3. Generation of *msnA* Disruption Strains of *A. parasiticus* and A. flavus

Preparation of protoplasts, *ptr*-based fungal transformation and selection of mutants were performed as previously described for *A. parasiticus* and *A. flavus* [14]. Homologous recombination is the primary event in fungi with the *ku70*-deficient background. The *msnA* gene disruption was confirmed by PCR with location-specific primers based on the expected genomic patterns generated by homologous recombination in the ∆ku70 genetic background. The primers used were P1: GACACAAGGTTCGTCGGTGACT and P2: GGTACTCGCGTCGCGATTA. PCR was carried out under the following conditions in a Perkin Elmer GeneAmp PCR System 2400. Twenty-five pmol of each primer and 10 ng genomic DNA were added to 25 μL Platinum Blue PCR Supermix (Invitrogen, Carlsbad, California, USA) and subject to 30 cycles consisting of denaturation at 94 °C for 30 s, annealing at 55 °C for 30 s and extension at 72 °C for 5 min.

### 2.4. Reintroduction of *msnA* into the *A. flavus* ∆msnA Strain

A genomic DNA fragment containing the full-length *msnA* gene including a 0.58 kb 5’UTR and a 0.24 kb 3’UTR was amplified by PCR using the Accuprime supermix (Invitrogen) with primers msn-E-pyrG, ATAGAATTCCCCGCGACTGTCCATTAGTC and msn-B-pyrG, ATAGGATCCTTTGTGAAGACCATGT. The PCR product was first cloned into the EcoR1 and BamH1 sites of pPG28 [18]. The *A. nidulans *autonomously-replicating sequence in the 5.2 kb HindIII fragment from pHELP1 [19] was cloned into the resulting construct. The circular plasmid was transformed into an *A. flavus* CA14∆msnA strain by the *pyrG*-based transformation protocol [15].

### 2.5. Colony Growth, Conidial Production, and Sclerotial Formation

The ∆msnA and the parental strains were point inoculated on PDA plates (100 × 15 mm) in triplicate and grown for five days at 30 °C for the determination of vegetative growth based on the colony diameter. For enumeration of conidia, two culture plugs were cored with Transfertube^®^ (Spectrum, Houston, Texas, USA) from each of the three seven-day-old replicate PDA plates. The plugs were placed in a microfuge tube containing 0.5 mL ethanol to kill conidia and then vortexed 2 min with a Disruption Genie apparatus (ZYMO RESEARCH, Orange County, California, USA). Each sample was diluted 100-fold with a 0.01% Triton solution, and conidia were counted two to four times using a hemocytometer. The calculated numbers were the total conidia from the two agar plugs. Strains were cultured on PDA and MCA plates at 30 °C for a week for sclerotial formation.

### 2.6. Ultraviolet-Visible (UV-Vis) Spectrophotometry and Fourier Transform Infrared (FTIR) Spectroscopy

The ∆msnA strains produced an unknown diffusible pigment on MCA plates not observed on PDA plates. The water-soluble orange pigment was isolated from 25 MCA plates inoculated with *A. parasiticus* BN9∆msnA. The frozen and thawed cultures were filtered through No. 4 paper (Whatman, Piscataway, New Jersey, USA) and the filtrate passed through a 33mm Millex GP 0.22 µ syringe filter (Millipore, Billerica, Massachusetts, USA). The filtrate was stirred overnight with Amberlite XAD-2 resin (Rohm and Haas, Philadelphia, Pennsylvania, USA). The pigment was eluted from the XAD-2 resin with MeOH, and the solvent was removed under reduced pressure. The remaining solids were dissolved in water and applied to a Sephadex G-25 column (GE Healthcare, Piscataway, New Jersey, USA). The column was eluted with water, and the orange band was collected and lyophilized yielding 44 mg of the pigment. The absorbance spectrum between 350 and 700 nm for the pigment dissolved in water was recorded on an Agilent 8453 photodiode array UV-Vis spectrophotometer (Agilent, Santa Clara, California, USA). Fourier Transform Infrared (FTIR) analysis, which provides information about the chemical bonding and the molecular structure of a compound, was performed. The infrared spectrum of the mixture of 0.5% of the orange pigment in KBr was obtained by diffuse reflectance on a Nicolet 6700 FTIR (Thermo Scientific, Waltham, Massachusetts, USA).

### 2.7. Determination of Aflatoxins and Kojic Acid by HPLC

The amounts of aflatoxins and kojic acid produced on MCA plates in triplicate by four- and eight-day-old cultures of wild type and ∆msn strains of *A. parasiticus *BN9 (only kojic acid was determined for RH), and *A. flavus *CA14 were determined. For each sample, the entire contents of the Petri dish (100 × 15 mm, 25 mL per plate) were added to a Waring MC-2 blender cup and blended for 30 s with 20 mL hot water. The mixture was filtered through Whatman No. 4 paper and a 33 mm Millipore Millex GP 0.22μm syringe filter. The filtered extracts were analyzed for aflatoxins and kojic acid on a HPLC system (Agilent) consisting of a quaternary pump, autosampler, photodiode array detector, and fluorescence detector. The analyses were performed on a column of Intersil ODS-3, 5 μ, 4.6 × 250 mm (GL Sciences, Torrance, California, USA) at a flow rate of 1.0 mL/min. The injection volume was 20 μL. The mobile phase for aflatoxins was H_2_O:CH_3_CN:MeOH (45:25:30) and for kojic acid MeOH:0.1% H_3_PO_4_ (25:75), isocratic. Detection of aflatoxins was based on fluorescence at 365 nm excitation and 455 nm emission with “PHRED” postcolumn photochemical derivatization (Aura Industries, New York, New York, USA). Detection of kojic acid was based on UV absorption at 265 nm. The retention times of the standards for aflatoxins B_1_, B_2_, G_1_ and G_2_ were 10.6 min, 9.4 min, 8.7 min and 7.8 min, respectively, and for kojic acid the retention time was 5.4 min.

### 2.8. RNA Isolation and Probe Labeling

An aliquot of spore suspension was added to each Petri dish plate (100 × 15 mm) containing 30 mL potato dextrose broth (PDB, Merck, Darmstadt, Germany) to give a final concentration of 3 × 10^5^ spores per milliliter. Four dishes were used for each strain. Stationary cultures were incubated at 30 °C in the dark for 4 days. Harvested mycelium were pooled and rinsed with sterilized distilled water and pulverized to a fine powder with a mortar and pestle in the presence of liquid nitrogen. Total RNA was prepared using TRIzol^®^ reagent (Invitrogen) and treated with amplification grade DNase I followed by purification with an RNeasy Plant Mini kit (Qiagen, Valencia, California, USA).

Fluorescent dye Cy3 and Cy5 labeled probes were prepared using the indirect labeling method of aminoallyl-cRNA according to the protocol provided by TIGR. A total of 6 μg of aminoallyl-cRNA were needed in each probe labeling. The aminoallyl-cRNA was synthesized and amplified using an Amino Allyl MessageAmp™ II aRNA Amplification kit (Ambion, Austin, Texas, USA). Mono-reactive dyes Cy3 and Cy5 (Amersham, Piscataway, New Jersey, USA) were coupled respectively to aminoallyl-cRNA from wild type and mutant. The unincorporated free dyes were removed using the RNeasy MinElute cleanup kit (Qiagen). 

### 2.9. Microarray Assays

The microarray used was *Aspergillus flavus* NRRL3357 27.6k oligo array [20]. A direct comparison design was applied, including Mutant/Wild type. Four technical replicates were used, including two dye swaps to compensate for cyanine dye effects. Following hybridization and washing according to the TIGR protocol, the microarray slides were scanned by a Genepix 4000B (Molecular Devices, Sunnyvale, California, USA), and the images were analyzed using GENEPIX 6.0 software. 

Microarray data were normalized and analyzed using GeneSpring GX 10.0 software (Silicon Genetics, Redwood City, California, USA). Two criteria were used for selecting positive spots, (Signal-Background) mean > 500 unit as expression intensity filter, and at least two of the four replicates showing positive. These filters were imposed to remove genes with very minor differential expression or genes with little evidence for expression. Data normalization was performed using local regression LOWESS (locally weighted scatterplot smoothing). Differentially expressed genes were identified by performing a one-way ANOVA on the normalized data using a T test with no assumption of equal variance. The cutoff criteria in fold change analysis were set as P < 0.05 in significant difference and fold change >2.

### 2.10. Quantitative PCR

Quantitative real-time PCR (qPCR) was carried out in a 20-μL reaction volume with the SYBR Green Master Mix (Applied Biosystems, Foster City, California, USA) in a StepOne^TM^ thermal cycler (Applied Biosystems). PCR conditions were as follows: an initial step of 95 °C for 10 min and 40 cycles with each cycle consisting of 95 °C for 15 s and 60 °C for 1 min. The primers sets used were designed by the PrimerExpress 3.0 software following the guidelines specified by Applied Biosystems. The specificity of the PCR products was confirmed by the melt curve analysis. The relative expression levels of the genes examined were determined by the relative standard curve method, in which standard curves based on 5-point, 10-fold serial dilutions were constructed for the endogenous (18S) and the target gene in each experiment using genomic DNA as the template.

### 2.11. Quantification of Reactive Oxygen Species (ROS)

ROS generation was assessed using the substrate 2’,7’-dichlorofluorescein diacetate (DCFH-DA; Sigma D6883). Oxidation of non-fluorescent DCFH-DA by ROS, such as H_2_O_2_ and the hydroxyl radical, yields the fluorescent product dichlorofluorescin (DCF). DCF fluorescence spectra is usually measured using excitation/emission wavelength of 490/525 nm. In this study, we used the OneStepPlus qPCR instrument (Applied Biosystems) that is able to measure dye fluorescence with the program set for the detection of SYBR^®^-DNA complex at excitation/emission wavelength of 488/522 nm. DCFH-DA stock solution (100×, 1 mM in dimethyl formamide) was prepared. Four agar plugs were cored from each PDA culture plate grown for 7 days at 30 °C. They were placed into a microfuge tube, and 1mL freshly made 1× PBS containing DCFH-DA at a final concentration of 1 μM was added. The reaction was allowed to continue at 37 °C for specified time periods (1, 5 or 24 hr). At the end of each time period, three replicates of 30 μL from each sample were loaded into the optical 48-well reaction plate. The program was set to hold at 37 °C and fluorescence measurements were taken for 6 cycles at 10 min intervals.

## 3. Results

### 3.1. Vegetative Growth, Conidial Production, and Sclerotial Formation of ∆msnA Strains

**Figure 1:**
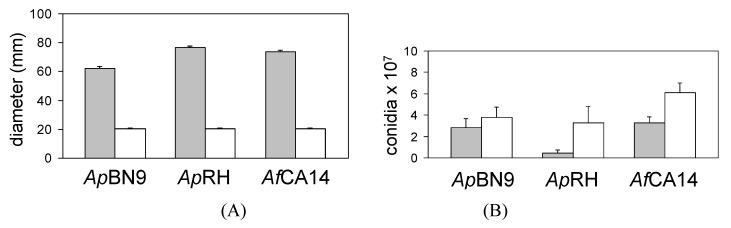
Effect of *msnA* disruption on colony size and conidial production. (A) Colony size after growth for five days at 30 °C. (B) Conidial production estimated from two cored agar plugs (see Materials and Methods 2.5). The gray bar represents the wild-type strain, and the clear bar represents the ∆msnA strain. Ap: *A. parasiticus*; Af: *A. flavus*.

**Figure S1:**
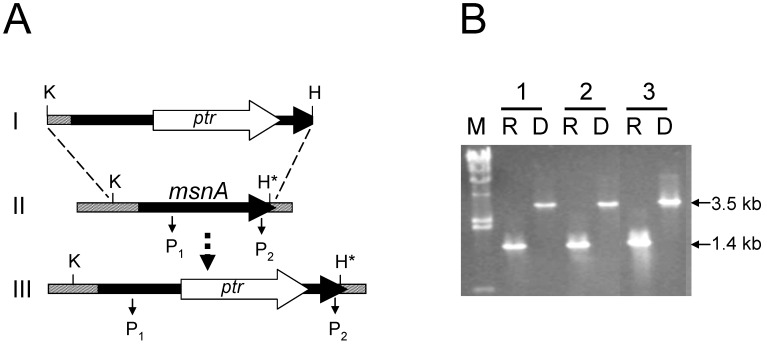
Disruption of the *msnA* gene in *A. parasiticus* and *A. flavus* by the *ptr* selectable marker. (A) Diagram depicting the gene disruption event via double-crossover recombination. I: linearized portion of the disruption vector; II: genomic pattern of the recipient strain; III: expected genomic pattern after recombination. (B) PCR confirmation of genomic DNA patterns of the recipient, R, and the *msnA* disruptant, D. The primers used were P1 and P2. The DNA size markers (in kb) are lambda DNA/Hind III fragments. The three sets are 1: *A. parasiticus* BN9, 2: *A parasiticus* RH and 3: *A. flavus* CA14.

Disruption of *msnA* in *A. parasiticus* BN9 and RH, and *A. flavus* CA14 was confirmed by PCR based on expected genomic patterns (Figure S1). Colonies of the ∆msnA strains of *A. parasiticus* and *A. flavus* exhibited a restricted, densely-packed appearance on PDA plates. The radial colony growth of the ∆msnA strains was 2 to 3-fold less than that of the parental strains (Figure 1A). Compared to the parental strains the ∆msnA strains produced more conidia as estimated from an identical area (Figure 1B). The increase in conidiation of *A. parasiticus* BN9∆msnA (36%) was less than that of *A. parasiticus* RH∆msnA (>600%) and *A. flavus* CA14∆msnA (84%). Although *A. parasiticus* RH is a strain that produces many sclerotia, RH∆msnA was unable to produce any on PDA; BN9∆msnA and CA14∆msnA were also unable to produce sclerotia on the same medium. 

**Figure 2:**
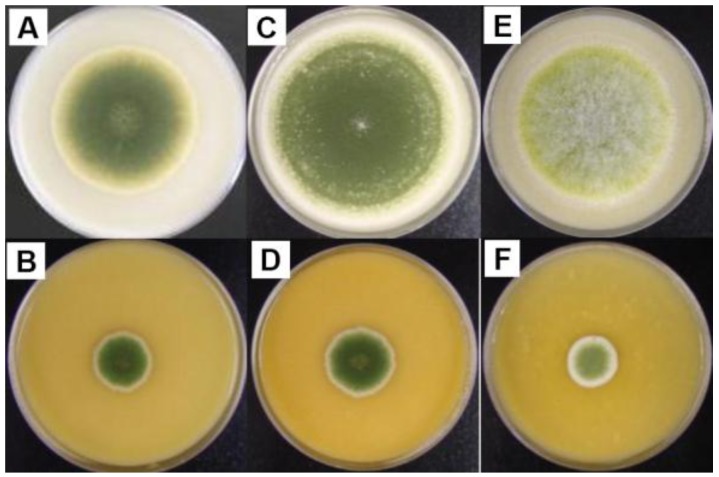
Culture morphology of *A. parasiticus* and *A. flavus* strains on MCA plates. (A) wild-type *A. parasiticus *BN9 (B) *A. parasiticus* BN9∆msnA (C) wild-type *A. parasiticus* RH; the white granules around the edge of the colony are sclerotia (D)*A. parasiticus* RH∆msnA (E) wild-type *A. flavus *CA14 (F) *A. flavus* CA14∆msnA.

To further examine whether sclerotial formation of the three ∆msnA strains was abolished, we grew them on mixed cereal agar plates. All strains did not produce sclerotia on this medium either. They, however, produced a diffusible orange-colored substance that increased in intensity after prolonged incubation (Figure 2). In contrast, the orange-colored pigment was barely produced by the wild-type strains after the same 7-day period of growth. 

### 3.2. Characterization of the Pigment by FTIR

**Figure 3:**
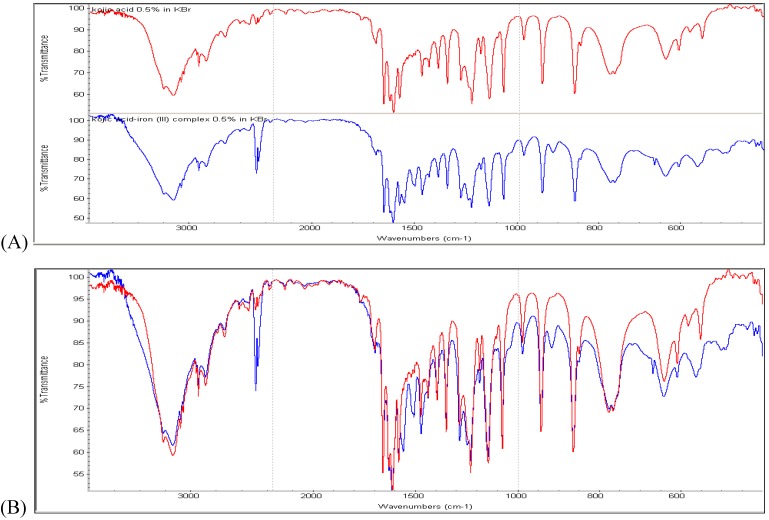
Characterization of the orange pigment by FTIR. (A) Spectrum of kojic acid: top panel red-line and spectrum of the pigment isolated from *A. parasiticus* BN9∆msnA: bottom panel blue-line. (B) Overlaid spectra.

**Figure S2:**
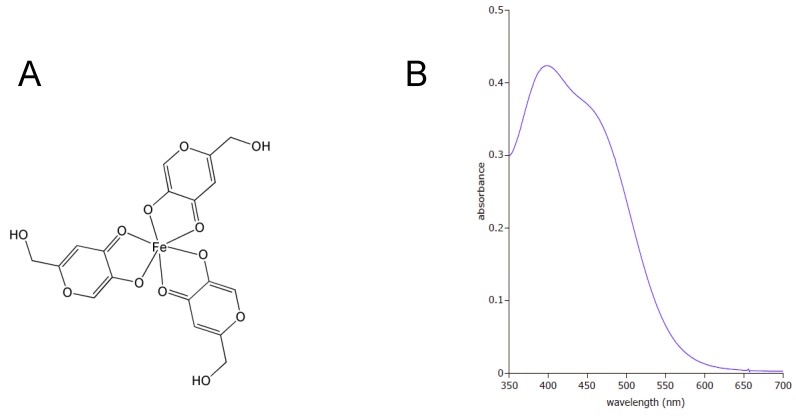
Characterization of the orange pigment by ultraviolet-visible spectrophotometry. (A) Structure of the 3kojic acid-ferric iron (Fe^+3^) complex. (B) UV-Vis spectrum of the orange pigment.

Based on the color we speculated that the orange pigment might be a complex derived from kojic acid, a metabolite commonly produced by some strains of *A. flavus* and closely related *A. oryzae*. The UV-Vis spectrum of the pigment purified from the *A. parasiticus* BN9∆msnA culture matched with the published spectrum of the complex of 3 kojic acid moieties per Fe(III) [21] and had a peak absorbance at 398 nm and a broad shoulder at 460 nm (Figure S2). The kojic acid in the complex was confirmed by FTIR analysis using kojic acid as the reference. The separate FTIR spectra of kojic acid and the pigment are shown in Figure 3A. The overlaid image indicates that the two spectra are nearly identical (Figure 3B); two extra peaks in the carbonyl region (1,500 to 1,700 cm^−1^) appear in the pigment and another observed difference is at about 2,550 cm^−1^. 

### 3.3. Production of Aflatoxins and Kojic Acid by the *∆msnA* Strains of *A. parasiticus* BN9 and A. flavus CA14

To determine how production of aflatoxins and kojic acid was affected by *msnA* disruption, we carried out a time-course quantitative determination. The normalized data (to growth area, Table 1) showed that the production of total aflatoxins and kojic acid increased from day 4 to day 8 in the *parasiticus* BN9∆msnA strain. The ∆msnA strain accumulated 50% more aflatoxins and 20-fold more kojic acid than the parental strain at day 8. The *A. parasiticus* strain RH is a blocked strain that accumulates *O*-methylsterigmatocystin as the end product. Although it does not produce aflatoxins, the derived RH∆msnA strain produced 10-fold more kojic acid. The *A. flavus* CA14∆msnA strain produced only small amounts of aflatoxins, but it produced four-fold more kojic acid than the parental strain.

**Table 1:** Production of aflatoxins and kojic acid by ∆msnA strains on MCA.

Strain(T)^a^	Colony(mm)^b^	AF(μg)^c^					KA(mg)^c^
		B_1_	B_2_	G_1_	G_2_	Total	
BN9(4)	42	2.87	0.09	4.50	0.09	7.58	0.06
BN∆msn (4)	17	4.36	0.13	5.11	0.09	9.69	1.06
BN9(8)	75	5.36	0.18	7.18	0.17	12.90	0.05
BN∆msn (8)	28	9.31	0.31	10.04	0.24	19.81	1.19
RH(4)	42					ND^d^	0.10
RH∆msn (4)	13					ND	1.17
RH(7)	67					ND	0.12
RH∆msn (8)	20					ND	1.38
CA14(4)	35	<0.01				<0.01	0.18
CA∆msn (4)	13	0.02				0.02	0.83
CA14(8)	76	<0.01				<0.01	0.35
CA∆msn (8)	27	0.01				0.01	1.33
a: T, days of growth. RH is a blocked strain and accumulates *O*-methylsterigmatocystin as the end product; it does not produce aflatoxins; b: Diameter averages from triplicate MCA plates; c: Data were normalized to an area with the radius of 1.0 cm; d: ND, not determined.							

### 3.4. Microarray Profiling of Differentially Expressed Genes in the ∆msnA Strains of *A. parasiticus* and *A. flavus*

Microarray assays identified many differentially expressed genes in the ∆msnA strains of *A. parasiticus* BN9 and *A. flavus *CA14 (Table S1 and Table S2). For *A. parasiticus*, approximately 85% of the genes (0.005% saturation and 500 cutoff) were down-regulated; only 12 genes were up-regulated (°2-fold), and they included those oxidative stress defense genes encoding superoxide dismutase, catalase, and cytochrome c peroxidase. The up-regulation of these genes was confirmed by qPCR (Table 2). For *A. flavus*, approximately two thirds of the genes profiled were down-regulated; the genes up-regulated were diverse including many of those encoding hypothetical proteins, and only one up-regulated catalase A gene for the *A. flavus* ∆msnA strain was found.

**Table 2:** Expression of oxidative stress defense genes in *A. parasiticus* BN9∆msnA.

Enzyme	Gene Locus	Oligoprimer Sequence	Fold-of-Increase ^c^
superoxide dismutase	AFL2G_10810.2 ^a^	cgccggtactgacgacctt	2.09 ° 0.06
	AFLA_099000 ^b^	agcattgccagtcttcttgga	
catalase	AFL2G_05806.2	caggtggcttcgcgtccta	2.30 ° 0.13
	AFLA_056170	caggccgcgcttcttg	
cytochrome c peroxidase	AFL2G_04481.2	tcggtcgtgcccatcct	2.38 ° 0.12
	AFLA_110690	aagacagtagggctgaagttcca	
Primers used for 18S rDNA in qPCR are ttcctagcgagcccaacct and cccgccgaagcaactaag.			
a: Broad Institute *Aspergillus* Comparative Database gene accession number (http://www.broadinstitute.org/annotation/genome/aspergillus_group/MultiHome.html); b: NCBI Entrez Gene accession number (http://www.ncbi.nlm.nih.gov/gene) (Table S1 and Table S2); c: Relative gene expression level ° S.D. The gene expression level of *A. parasiticus *BN9 is 1.00.			

### 3.5. ROS Production by *A. parasiticus* and *A. flavus* ∆msnA Strains and by *A. flavus msnA* Addback Strains

**Figure S3:**
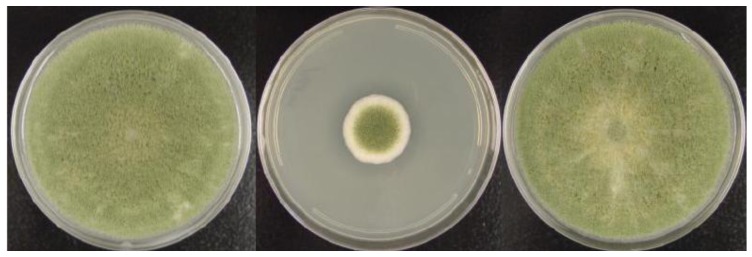
Colony morphology of *A. flavus* CA14∆msnA retransformed with the genomic *msnA* gene. Left: wild type, middle: CA14∆msnA, and right: msnA addback transformant. Cultures were grown at 30 °C for a week in the dark on PDA plates.

The recent generation of the *A. flavus* CA14-derived double mutant makes it possible to retransform a knockout strain, such as the ∆msnA strain, using a second selectable marker along with an intact gene to confirm gene function [15]. By this approach, the defects in colony growth were remediated in the *A. flavus* CA14∆msnA strain after the intact *msnA* genomic DNA was reintroduced (Figure S3). 

**Figure 4:**
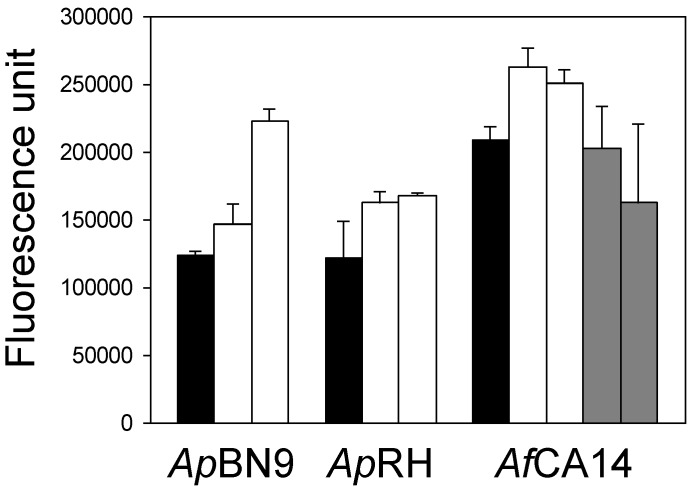
Production of reactive oxygen species (ROS) by *A. parasiticus* and *A. flavus *strains. Solid bar: parental strain, clear bar: independent ∆msnA strains, and gray bar, independent msnA addback strains.

ROS production by the wild-type strains, ∆msnA strains of *A. parasiticus* and *A. flavus*, and by the *A. flavus *msnA addback strain was determined to see whether ROS levels correlated with observed changes in growth, development and secondary metabolite production. ROS production was found to fluctuate in the first 5 hr incubation assays (data not shown). Only after 24 hr incubation a more distinct pattern emerged; an increase in ROS production was seen in all ∆msnA strains (Figure 4). ROS production by the *A. flavus* msnA addback strains was decreased to levels comparable to that of the wild-type *A. flavus*. 

## 4. Discussion

Our results show that *msnA* is required for normal colony growth and development of *A. parasiticus* and *A. flavus*. Similar findings have been reported for *Trichoderma atrovidire* [4] and *A. nidulans* [22]. The role of oxidative stress in conidiation has been implicated in *A. nidulans* [23]. Likewise, exposure of *Neurospora crassa* to antioxidants inhibits conidiation [24]. The *N. crassa* catalase-3 gene mutant that was sensitive to hydrogen peroxide produced six-times more conidia [25]. The increased production of conidia in the ∆msnA strains (Figure 1B) can be correlated with the increased levels of ROS (Figure 4) and suggests that conidiation is a response to increased levels of oxidative stress in the cells. 

Sclerotial formation like conidiation has been correlated with oxidative stress, antioxidant effects, and antioxidant enzyme activities [26,27]. Sclerotia of *A. parasiticus* and *A. flavus* are considered to be a vestige of the sexual cleistothecia produced by other aspergilli [28]. The light responses of the two structures regulated by the common factor VeA [29,30,31] are similar. In the dark *A. nidulans* favors the formation of cleistothecia [32,33], and under light *A. parasiticus* and *A. flavus* suppress sclerotial production [34]. The ∆msnA strains of *A. parasiticus* and *A. flavus* are unable to produce sclerotia. This result was unexpected since the *A. nidulans nrdA*(*msnA*)-null strain produced enhanced levels of cleistothecia [22]. It suggests that subtle differences likely exist in the regulation of sclerotial and cleistothecial morphogenesis. Impairment of *msnA* like the disruption of *laeA*, a secondary metabolism regulatory gene [35], abolishes sclerotial formation but not conidiation, which shows that the two developmental processes are regulated differently.

Aflatoxin biosynthesis may be a defense mechanism against oxidative stress [36,37,38]. Studies have demonstrated that natural antioxidants, such as gallic acid, caffeic acid and eugenol can reduce aflatoxin production [39,40,41]. Several lines of evidence also have suggested a positive correlation between ROS accumulation and aflatoxin production by *A. flavus* and *A. parasiticus* [36,42]. Deletion of the *A. parasiticus yapA* gene, which encodes a transcription factor that mediates oxidative stress response, resulted in precocious ROS formation and increased aflatoxin biosynthesis [38]. Supporting this correlation we found that compared to respective parental strains, the ∆msnA strains produced more aflatoxins (Table 1) and had higher levels of ROS accumulation (Figure 4). Beside aflatoxins, FTIR assays confirm that the orange pigment is a kojic acid-iron complex (Figure 3). The peaks at 1500 and 1580 cm^−1^ in the spectrum of the pigment are consistent with the chelation of kojic acid with a metal through the carbonyl moiety (Figure 3B). Kojic acid is a scavenger of free radicals [43]. The highly elevated production levels (Table 1) suggest that the ∆msnA strains use the formation of kojic acid as a main detoxifying mechanism.

Microarray comparisons of ∆msnA to wild-type showed that genes encoding superoxide dismutase, catalase, and cytochrome c peroxidase in the *A. parasiticus* BN9∆msnA strain were up-regulated (Table 2). The result suggests that expression of these genes is probably needed to cope with increased oxidative stress in the cells. The generation of ROS is potentially deleterious. Superoxide dismutase converts superoxide to another ROS, hydrogen peroxide, probably to shunt the superoxide away from harmful lipid peroxidation to the cells [44] or from damages to DNA [45]. Catalase converts hydrogen peroxide to water and oxygen molecule. Like catalase, cytochrome c peroxidase takes reducing equivalents from cytochrome c and converts hydrogen peroxide to water. Different types of oxidative stress defense genes operate in the ∆msnA strains of *A. parasiticus* and *A. flavus*; this likely reflects respective physiological responses of each species in spite of their close genetic relatedness. The varied amounts of aflatoxins and/or kojic acid produced by respective ∆msnA strains also reflect intrinsic differences of the two species and within members of the same species, such as BN9 and RH. 

## 5. Conclusions

This study suggests that the ∆msnA strains of *A. parasiticus* and *A. flavus* are not as capable as the wild-type strains in relieving oxidative stress and respond by up-regulating antioxidant enzyme genes as well as by increasing the production of conidia, aflatoxins, and kojic acid to alleviate the increased oxidative stress caused by the loss of *msnA*.

## Supplementary Materials

**Table S1:** Genes differentially expressed in *A. parasiticus *BN9∆msnA.

Gene ID (a)	Fold change	Regulation	Annotation
AFLA_003050	2.13	down	RTA1 like protein
AFLA_003050	2.12	down	RTA1 like protein
AFLA_003090	2.10	down	hypothetical protein
AFLA_004690	2.12	up	hypothetical protein
AFLA_004690	2.21	up	hypothetical protein
AFLA_004940	8.07	down	Glycolipid 2-alpha-mannosyltransferase family protein
AFLA_005520	3.38	down	hypothetical protein
AFLA_006160	2.90	down	hypothetical protein
AFLA_011970	2.22	down	Major Facilitator Superfamily protein
AFLA_014260	3.08	up	hydrophobin, putative
AFLA_014960	2.73	down	hypothetical protein
AFLA_015300	2.03	down	FMN-dependent dehydrogenase family protein
AFLA_015300	2.43	down	FMN-dependent dehydrogenase family protein
AFLA_015550	4.26	down	Sugar transporter family protein
AFLA_015780	5.24	down	small oligopeptide transporter, OPT family protein
AFLA_017860	2.00	up	hypothetical protein
AFLA_024250	2.01	up	Amidase family protein
AFLA_027340	2.04	down	Aha1 domain family, putative
AFLA_027340	2.16	down	Aha1 domain family, putative
AFLA_028030	3.03	down	conserved hypothetical protein
AFLA_029390	4.22	down	HMG box family protein
AFLA_036040	2.18	down	mitochondrial inner membrane translocase subunit (TIM17), putative
AFLA_036040	2.23	down	mitochondrial inner membrane translocase subunit (TIM17), putative
AFLA_036980	2.76	down	MOSC N-terminal beta barrel domain containing protein
AFLA_037160	2.40	up	thiazole biosynthesis enzyme, putative
AFLA_037160	2.29	up	thiazole biosynthesis enzyme, putative
AFLA_037290	2.10	down	hypothetical protein
AFLA_037290	5.14	down	hypothetical protein
AFLA_040140	2.02	down	Major intrinsic protein
AFLA_040400	6.62	down	hypothetical protein
AFLA_041930	2.93	down	conserved hypothetical protein
AFLA_042970	3.98	down	MIF4G domain containing protein
AFLA_042970	5.44	down	MIF4G domain containing protein
AFLA_046230	4.20	down	amino acid permease (Dip5), putative
AFLA_046400	4.57	down	unknown-related
AFLA_046400	4.41	down	DUF788 domain protein
AFLA_051570	3.88	down	To ribosomal protein YmL36 precursormitochondrial
AFLA_052640	2.07	down	PH domain containing protein
AFLA_056170	2.10	up	mycelial catalase Cat1, putative
AFLA_056170	2.05	up	mycelial catalase Cat1, putative
AFLA_056480	2.91	down	glycosyl transferase, group 2 family protein
AFLA_056480	3.85	down	glycosyl transferase, group 2 family protein
AFLA_057950	2.50	up	hypothetical protein
AFLA_057950	2.33	up	hypothetical protein
AFLA_059660	2.38	down	Major intrinsic protein
AFLA_060050	3.07	down	Amino acid permease family protein
AFLA_060050	3.99	down	Amino acid permease family protein
AFLA_060090	3.87	down	Major Facilitator Superfamily protein
AFLA_068610	2.92	down	hypothetical protein
AFLA_070900	3.43	down	hypothetical protein
AFLA_073580	2.96	down	cell division control protein Cdc6, putative
AFLA_073800	2.07	up	short chain dehydrogenase/reductase family, putative
AFLA_073800	2.10	up	short chain dehydrogenase/reductase family, putative
AFLA_076860	2.38	down	MOSC domain containing protein
AFLA_080910	2.98	down	hypothetical protein
AFLA_080910	2.65	down	hypothetical protein
AFLA_085250	2.03	down	RNA recognition motif. (a.k.a. RRM, RBD, or RNP domain) protein, putative
AFLA_087740	3.09	down	ANTH domain containing protein
AFLA_089240	2.98	down	Amidase family protein
AFLA_089240	3.09	down	Amidase family protein
AFLA_091600	3.23	down	nascent polypeptide-associated complex (NAC) subunit, putative
AFLA_091980	4.26	down	Ctr copper transporter family protein
AFLA_091980	3.38	down	Ctr copper transporter family protein
AFLA_092600	2.54	down	hypothetical protein
AFLA_094470	2.58	down	To UV radiation resistance associated protein p63
AFLA_096520	2.11	down	hypothetical protein
AFLA_096570	4.88	down	hypothetical protein
AFLA_097790	2.15	down	To chloride-bicarbonate anion exchanger AE2, putative
AFLA_098050	3.28	down	gamma-cysteine synthetase regulatory subunit, putative
AFLA_099000	2.07	up	Cu, Zn superoxide dismutase SOD1, putative
AFLA_106370	2.46	down	Conserved hypothetical ATP binding protein
AFLA_110690	2.04	up	Peroxidase family protein
AFLA_110690	2.03	up	Peroxidase family protein
AFLA_111740	2.83	down	To SAC1 protein, putative
AFLA_111740	3.30	down	To SAC1 protein, putative
AFLA_112180	2.50	down	ATP-dependent RNA helicase, putative
AFLA_112420	2.59	down	hypothetical protein
AFLA_112540	4.63	down	hypothetical protein
AFLA_112540	5.17	down	hypothetical protein
AFLA_114570	3.92	down	conserved hypothetical protein
AFLA_118050	2.31	down	POT family protein
AFLA_118050	2.47	down	POT family protein
AFLA_119430	2.07	down	Sec1 family protein
AFLA_120960	2.50	down	hypothetical protein
AFLA_122110	2.32	up	bifunctional catalase-peroxidase Cat2
AFLA_123290	3.12	down	hypothetical protein
AFLA_123290	2.35	down	hypothetical protein
AFLA_124420	2.32	down	amine oxidase, flavin-containing family protein
AFLA_127570	3.63	down	hypothetical protein
AFLA_128560	3.84	down	PrnA protein, putative
AFLA_131410	3.93	down	PCI domain containing protein
AFLA_132310	2.45	down	tRNA intron endonuclease, catalytic C-terminal domain containing protein
AFLA_132310	3.58	down	tRNA intron endonuclease, catalytic C-terminal domain containing protein
AFLA_132750	2.47	down	conserved hypothetical protein
AFLA_133810	3.05	down	conserved hypothetical protein
AFLA_137510	4.23	down	Emopamil binding protein
AFLA_138590	2.01	up	cysteine-type peptidase, putative
AO090012000538	2.90	down	predicted protein
AO090012000538	2.45	down	predicted protein
(a) NCBI Entrez Gene ( http://www.ncbi.nlm.nih.gov/gene) accession number.			

**Table S2:** Genes differentially expressed in *A. flavus *CA14∆msnA.

Gene ID	Fold change	Regulation	Annotation
AFLA_001010	4.06	up	hypothetical protein
AFLA_002840	3.43	down	conserved hypothetical protein
AFLA_002840	4.83	down	conserved hypothetical protein
AFLA_002860	2.62	down	Oxidoreductase family, NAD-binding Rossmann fold containing protein
AFLA_002940	2.43	down	Glycosyl hydrolase family 76 protein
AFLA_003980	2.51	down	cation diffusion facilitator family transporter containing protein
AFLA_003980	2.38	down	cation diffusion facilitator family transporter containing protein
AFLA_004420	2.22	up	hypothetical protein
AFLA_007830	2.85	up	hypothetical protein
AFLA_007830	3.00	up	hypothetical protein
AFLA_007860	2.09	up	Major Facilitator Superfamily protein
AFLA_007860	2.12	up	Major Facilitator Superfamily protein
AFLA_010180	2.42	up	hypothetical protein
AFLA_010180	2.35	up	hypothetical protein
AFLA_011370	2.17	up	hypothetical protein
AFLA_011370	2.23	up	hypothetical protein
AFLA_011530	5.11	up	hypothetical protein
AFLA_011530	5.76	up	hypothetical protein
AFLA_011560	2.49	up	Phosphoesterase family protein
AFLA_011560	2.43	up	Phosphoesterase family protein
AFLA_012030	2.10	down	DUF895 domain membrane protein, putative
AFLA_012030	2.08	down	DUF895 domain membrane protein, putative
AFLA_012050	2.68	down	*N*-acetylglucosamine-6-phosphate deacetylase family protein
AFLA_012050	2.47	down	*N*-acetylglucosamine-6-phosphate deacetylase family protein
AFLA_012080	2.74	down	glucosamine-6-phosphate deaminase, putative
AFLA_012080	2.74	down	glucosamine-6-phosphate deaminase, putative
AFLA_013060	2.88	down	expressed protein, putative
AFLA_013060	2.83	down	expressed protein, putative
AFLA_013740	2.46	up	acid phosphatase SurE family protein
AFLA_013740	2.45	up	acid phosphatase SurE family protein
AFLA_014210	3.22	down	Major Facilitator Superfamily protein
AFLA_014210	3.78	down	Major Facilitator Superfamily protein
AFLA_014510	4.89	down	Formate/nitrite transporter family protein
AFLA_014510	4.33	down	Formate/nitrite transporter family protein
AFLA_015780	4.29	down	small oligopeptide transporter, OPT family protein
AFLA_015780	3.91	down	small oligopeptide transporter, OPT family protein
AFLA_015800	2.25	down	conserved hypothetical protein
AFLA_017750	3.37	down	conserved hypothetical protein
AFLA_017750	2.75	down	conserved hypothetical protein
AFLA_018790	3.16	down	nitrate transporter (nitrate permease), putative
AFLA_018790	3.21	down	nitrate transporter (nitrate permease), putative
AFLA_019510	2.60	up	conserved hypothetical protein
AFLA_019510	3.04	up	conserved hypothetical protein
AFLA_021000	2.16	down	conserved hypothetical protein
AFLA_022370	2.08	down	hypothetical protein
AFLA_023610	3.94	down	hypothetical protein
AFLA_025130	4.27	up	To blastomyces yeast phase-specific protein 1
AFLA_025960	2.26	down	Nucleoside transporter family protein
AFLA_025960	2.10	down	Nucleoside transporter family protein
AFLA_026950	2.05	down	acetyl-CoA-acetyltransferase, putative
AFLA_026950	2.22	down	acetyl-CoA-acetyltransferase, putative
AFLA_028830	2.30	up	FG-GAP repeat family protein
AFLA_028830	2.45	up	FG-GAP repeat family protein
AFLA_028950	2.41	down	Glycosyl hydrolase family 81 protein
AFLA_029000	2.03	up	hypothetical protein
AFLA_029000	2.05	up	hypothetical protein
AFLA_029970	3.69	down	conserved hypothetical protein
AFLA_029970	3.51	down	conserved hypothetical protein
AFLA_031380	3.64	down	class V chitinase, putative
AFLA_031380	3.82	down	class V chitinase, putative
AFLA_034140	3.10	down	Major Facilitator Superfamily protein
AFLA_034140	2.88	down	Major Facilitator Superfamily protein
AFLA_036370	2.37	down	phosphoenolpyruvate carboxykinase (ATP), putative
AFLA_036370	2.24	down	phosphoenolpyruvate carboxykinase (ATP), putative
AFLA_037820	3.75	up	Hsp20/alpha crystallin family protein
AFLA_037820	3.81	up	Hsp20/alpha crystallin family protein
AFLA_040140	2.07	down	Major intrinsic protein
AFLA_040330	4.54	down	Chitin binding Peritrophin-A domain containing protein
AFLA_040330	4.58	down	Chitin binding Peritrophin-A domain containing protein
AFLA_041010	2.16	up	hypothetical protein
AFLA_041010	2.16	up	hypothetical protein
AFLA_041180	7.25	down	hypothetical protein
AFLA_042000	2.01	down	D-isomer specific 2-hydroxyacid dehydrogenase family protein, putative
AFLA_042360	2.07	up	hypothetical protein
AFLA_042360	2.01	up	hypothetical protein
AFLA_042540	2.38	up	hypothetical protein
AFLA_043390	2.26	down	hypothetical protein
AFLA_043390	2.13	down	hypothetical protein
AFLA_044040	3.66	down	hypothetical protein
AFLA_044720	3.39	down	permease, cytosine/purines, uracil, thiamine, allantoin family protein
AFLA_044720	3.37	down	permease, cytosine/purines, uracil, thiamine, allantoin family protein
AFLA_046620	2.91	up	MAPEG family protein
AFLA_046620	2.66	up	MAPEG family protein
AFLA_049470	3.22	up	hypothetical protein
AFLA_049470	3.36	up	hypothetical protein
AFLA_050070	2.19	down	conserved hypothetical protein
AFLA_050940	2.08	down	phenylalanyl-tRNA synthetase, beta subunit, putative
AFLA_053700	2.07	up	hypothetical protein
AFLA_055550	2.75	down	conserved hypothetical protein
AFLA_055550	2.55	down	conserved hypothetical protein
AFLA_058030	2.77	down	MFS transporter, putative
AFLA_058030	2.81	down	MFS transporter, putative
AFLA_060260	2.32	up	heat shock protein HSP30, putative
AFLA_062460	2.46	down	non-classical export protein (Nce2), putative
AFLA_062460	2.66	down	non-classical export protein (Nce2), putative
AFLA_063260	3.03	down	Sic1.20-related
AFLA_063260	3.08	down	Sic1.20-related
AFLA_063290	3.92	down	hypothetical protein
AFLA_063290	4.09	down	hypothetical protein
AFLA_063320	3.34	down	hypothetical protein
AFLA_063320	3.74	down	hypothetical protein
AFLA_065220	4.99	up	hypothetical protein
AFLA_065220	4.93	up	hypothetical protein
AFLA_065450	3.37	down	Deuterolysin metalloprotease, putative
AFLA_065450	3.01	down	Deuterolysin metalloprotease, putative
AFLA_065460	6.03	down	hypothetical protein
AFLA_065460	7.02	down	hypothetical protein
AFLA_065960	3.05	up	fucose-specific lectin, putative
AFLA_065960	3.02	up	fucose-specific lectin, putative
AFLA_066810	4.31	up	To blastomyces yeast phase-specific protein 1
AFLA_067640	2.15	down	alternative NADH-dehydrogenase, putative
AFLA_067640	2.18	down	alternative NADH-dehydrogenase, putative
AFLA_067770	2.62	down	PQ loop repeat family protein
AFLA_067770	2.64	down	PQ loop repeat family protein
AFLA_068600	2.89	down	ammonium transporter MEAA, putative
AFLA_068600	2.90	down	ammonium transporter MEAA, putative
AFLA_068790	2.23	down	adenylylsulfate kinase, putative
AFLA_068790	2.27	down	adenylylsulfate kinase, putative
AFLA_070070	2.09	up	hypothetical protein
AFLA_070070	2.07	up	hypothetical protein
AFLA_070470	2.02	up	hypothetical protein
AFLA_074060	2.40	down	R3H domain containing protein
AFLA_075190	2.96	down	conserved hypothetical protein
AFLA_075190	2.94	down	conserved hypothetical protein
AFLA_078210	2.37	down	membrane protein-related
AFLA_078210	2.36	down	membrane protein-related
AFLA_078900	3.11	down	Glycosyl hydrolase family 20, catalytic domain containing protein
AFLA_078900	2.70	down	Glycosyl hydrolase family 20, catalytic domain containing protein
AFLA_083890	2.60	up	oxidoreductase, zinc-binding dehydrogenase family protein
AFLA_083890	2.59	up	oxidoreductase, zinc-binding dehydrogenase family protein
AFLA_087630	2.96	down	alpha, alpha-trehalose-phosphate synthase subunit, putative
AFLA_087630	2.36	down	alpha, alpha-trehalose-phosphate synthase subunit, putative
AFLA_087750	2.96	down	isopentenyl-diphosphate delta-isomerase, putative
AFLA_090690	2.25	up	catalase A, putative
AFLA_090690	2.73	up	catalase A, putative
AFLA_090970	2.24	down	conserved hypothetical protein
AFLA_090970	2.09	down	conserved hypothetical protein
AFLA_091260	2.08	down	acetyltransferase, GNAT family protein
AFLA_091260	2.15	down	acetyltransferase, GNAT family protein
AFLA_094630	2.11	down	hypothetical protein
AFLA_094630	2.16	down	hypothetical protein
AFLA_095460	2.39	down	PBS lyase HEAT-like repeat family protein
AFLA_098380	3.39	down	conidial hydrophobin RodA, putative
AFLA_098380	3.77	down	conidial hydrophobin RodA, putative
AFLA_098700	2.54	down	oxidoreductase, short chain dehydrogenase/reductase family protein
AFLA_098700	2.47	down	oxidoreductase, short chain dehydrogenase/reductase family protein
AFLA_099050	3.83	down	hypothetical protein
AFLA_099050	3.70	down	hypothetical protein
AFLA_101780	2.41	down	Oxidoreductase molybdopterin binding domain containing protein
AFLA_101780	2.20	down	Oxidoreductase molybdopterin binding domain containing protein
AFLA_101800	3.81	down	Glycosyl hydrolases family 18 protein
AFLA_101800	4.33	down	Glycosyl hydrolases family 18 protein
AFLA_104350	8.01	down	Dynamin central region family protein
AFLA_104350	6.90	down	Dynamin central region family protein
AFLA_105630	3.94	up	Cytochrome P450 family protein
AFLA_105630	3.82	up	Cytochrome P450 family protein
AFLA_109030	3.01	down	To nucleotide exsicion repair protein RAD7
AFLA_109160	3.31	down	isopentenyl-diphosphate delta-isomerase, putative
AFLA_110040	5.26	down	blr7677-related
AFLA_110040	6.41	down	blr7677-related
AFLA_112720	2.26	down	diphosphomevalonate decarboxylase, putative
AFLA_112910	2.85	up	hypothetical protein
AFLA_112910	2.89	up	hypothetical protein
AFLA_113790	2.78	down	hypothetical protein
AFLA_113790	2.39	down	hypothetical protein
AFLA_115930	3.40	up	hypothetical protein
AFLA_115930	3.22	up	hypothetical protein
AFLA_119340	2.12	up	Helix-loop-helix DNA-binding domain containing protein
AFLA_119340	2.29	up	Helix-loop-helix DNA-binding domain containing protein
AFLA_125770	3.03	down	hypothetical protein
AFLA_125770	3.25	down	hypothetical protein
AFLA_127620	7.70	down	New cDNA-based gene: (AO_CDS_042706, novel, updateIDs: 11597, [gene: novel_gene_1223, model: novel_model_1223])
AFLA_127620	11.51	down	New cDNA-based gene: (AO_CDS_042706, novel, updateIDs: 11597, [gene: novel_gene_1223, model: novel_model_1223])
AFLA_129810	3.08	down	cytoplasmic asparaginyl-tRNA synthetase, putative
AFLA_129810	2.94	down	cytoplasmic asparaginyl-tRNA synthetase, putative
AFLA_130150	2.02	down	NAD+ dependent glutamate dehydrogenase, putative
AFLA_133830	2.02	down	oxidoreductase, zinc-binding dehydrogenase family protein
AFLA_134420	2.02	up	Sugar transporter family protein
AFLA_139270	2.28	up	aflNa/ hypD/ hypothetical protein
AFLA_139270	2.37	up	aflNa/ hypD/ hypothetical protein
AFLA_139290	3.06	up	aflMa/ hypE/ hypothetical protein
AFLA_139400	2.17	up	aflCa/hypC/hypothetical protein
AFLA_139400	2.03	up	aflCa/hypC/hypothetical protein
AO090120000447	6.76	down	predicted protein
AO090120000447	5.18	down	predicted protein
a: NCBI Entrez Gene ( http://www.ncbi.nlm.nih.gov/gene) accession number.			
